# Age-Related Trends in the Trabecular Micro-Architecture of the Medial Clavicle: Is It of Use in Forensic Science?

**DOI:** 10.3389/fbioe.2019.00467

**Published:** 2020-01-22

**Authors:** Hannah McGivern, Charlene Greenwood, Nicholas Márquez-Grant, Elena F. Kranioti, Bledar Xhemali, Peter Zioupos

**Affiliations:** ^1^Cranfield Forensic Institute, Cranfield University, Defence Academy of the United Kingdom, Shrivenham, United Kingdom; ^2^School of Chemistry and Physical Sciences, Keele University, Keele, United Kingdom; ^3^Edinburgh Unit for Forensic Anthropology, School of History Classics and Archaeology, University of Edinburgh, Edinburgh, United Kingdom; ^4^Forensic Medicine Unit, Department of Forensic Sciences, Faculty of Medicine, University of Crete, Heraklion, Greece; ^5^Institute of Forensic Medicine, Tirana, Albania

**Keywords:** clavicle, cancellous bone, aging, micro-computed tomography, forensic science, biomechanics

## Abstract

The mechanical and structural properties of bone are known to change significantly with age. Within forensic and archaeological investigations, the medial end of the clavicle is typically used for estimating the age-at-death of an unknown individual. Although, this region of the skeleton is of interest to forensic and clinical domains, alterations beyond the macro-scale have not been fully explored. For this study, non-destructive micro-computed tomography (μ-CT) was employed to characterize structural alterations to the cancellous bone of the medial clavicle. Fresh human cadaveric specimens (12-59 years) obtained at autopsy were utilized for this study, and were scanned with a voxel size of ~83 μm. Morphometric properties were quantified and indicated that the bone volume, connectivity density, mineral density, and number of trabeculae decreased with age, while the spacing between the trabeculae increased with age. In contrast to other sub-regions of the skeleton, trabecular thickness, and degree of anisotropy did not correlate with age. Collectively, this could suggest that the network is becoming increasingly perforated with age rather than exhibiting trabecular thinning. These results are used in the context of deriving a potential protocol for forensic investigations by using this particular and largely unexplored region of the skeleton, and provide inspiration for future experiments concerning micro-architectural and small scale changes in other regions of the human skeleton.

## Introduction

The dynamic and heterogeneous nature of bone is an engineering feat of human evolution. This biological composite is designed to achieve an optimal balance between lightness, strength and toughness, allowing the skeleton to perform its primary mechanical functions (Martin, 1993; Zhou et al., 1996; Beck and Marcus, 1999; Currey, 2002; Tranquilli Leali et al., 2009; Chen et al., 2013; Johannesdottir and Bouxsein, 2018). Bone's intricate and sophisticated design is evident through the hierarchical levels of its structure and maintains homeostasis by balancing repair and break down (Martin, 1993; Zhou et al., 1996; Rho et al., 1998; Beck and Marcus, 1999; Compston, 2011; Johannesdottir and Bouxsein, 2018). With aging the balance is upset in favor of bone break down resulting in a decline in bone strength and inner deterioration of the material structurally, physically, and mechanically (Zhou et al., 1996; Beck and Marcus, 1999; Currey, 2002).

Degenerative changes often manifest primarily in shape and form at the macro-scale, and these biomarkers have been utilized in the field of forensic anthropology for developing methods and models with which to estimate the age-at-death of an unknown individual. Typically, certain sub-regions of the human skeleton such as the pelvis, sternal end of the fourth rib and medial clavicle are matched with a “score” or “phase” from an established classification system (Schmeling et al., 2004; Franklin, 2010; Kellinghaus et al., 2010). It has been customary to use the clavicle, which provides information for estimating age-at-death when other elements of the skeleton are fused, because of its protracted maturation period (Schmeling et al., 2004; Kellinghaus et al., 2010; Milenkovic et al., 2013; Wittschieber et al., 2014, 2016; LeBel, 2015; Rudolf et al., 2018; Doyle et al., 2019). There has been a gradual transition to radiographic imaging modalities but established scoring systems continue to be implemented (Schmeling et al., 2004; Kellinghaus et al., 2010; Wittschieber et al., 2014, 2016; Rudolf et al., 2018). Scoring systems, as a method, are subjective and rely on the expertise and experience of the forensic examiner (Zioupos et al., 2014), and can be foiled by other factors such as soft tissue attachments which obscure the true topography of the articular surface needed for accurate assessment (Crowder and Pfeiffer, 2010).

The inner micro-structure of the clavicle has not been examined at length and in the forensic context it may lead to the development of innovative and quantitative analytical methods. Such information can be acquired through the application of micro-computed tomography (μ-CT), a non-destructive imaging technique which has been used previously to quantify structural alterations to the network of trabeculae mostly in the epiphyses of long bones and vertebral bodies implicated for the increased risk of fracture with age (Müller and Rüegsegger, 1997; Müller et al., 1998; Hildebrand et al., 1999; Ulrich et al., 1999; Eckstein et al., 2007; Chen et al., 2008, 2010, 2013; Cui et al., 2008; Lochmüller et al., 2008; Djuric et al., 2010; Viguet-Carrin et al., 2010; Thomsen et al., 2013, 2015; Vale et al., 2013; Greenwood et al., 2018; Whitmarsh et al., 2019). The objectives of this study were to examine the inner structure of the medial clavicle by μ-CT, in order to characterize changes in the trabecular micro-structure as a function of age as well as examine the implications and potential input of this work in future case studies on aging providing a more objective analytical basis for age estimation in forensic science.

## Materials and Methods

Human cadaveric samples (12–59 years) of 24 right clavicles dissected at autopsy (18 M/6 F) were provided by the forensic institute in Tirana, Albania. Ethical approval was provided by Cranfield University Ethics Committee (CURES/2294/2017), the respective governing bodies from Albania (the country of origin) and NHS tissue governance audit (ICA01/17). The cause of death was mostly sudden or accidental (forensic cases), but not health related such that could affect the condition of the bone samples. Additional information pertaining to the body weight and lifestyle of each donor was not available, although these confounding variables can affect the rate of skeletal change associated with age and morphology at the macro-scale. All specimens were securely stored at −20°C before and between testing. All samples were anonymised prior to acquisition.

### μ-CT Imaging Protocol

**Figure 1:**
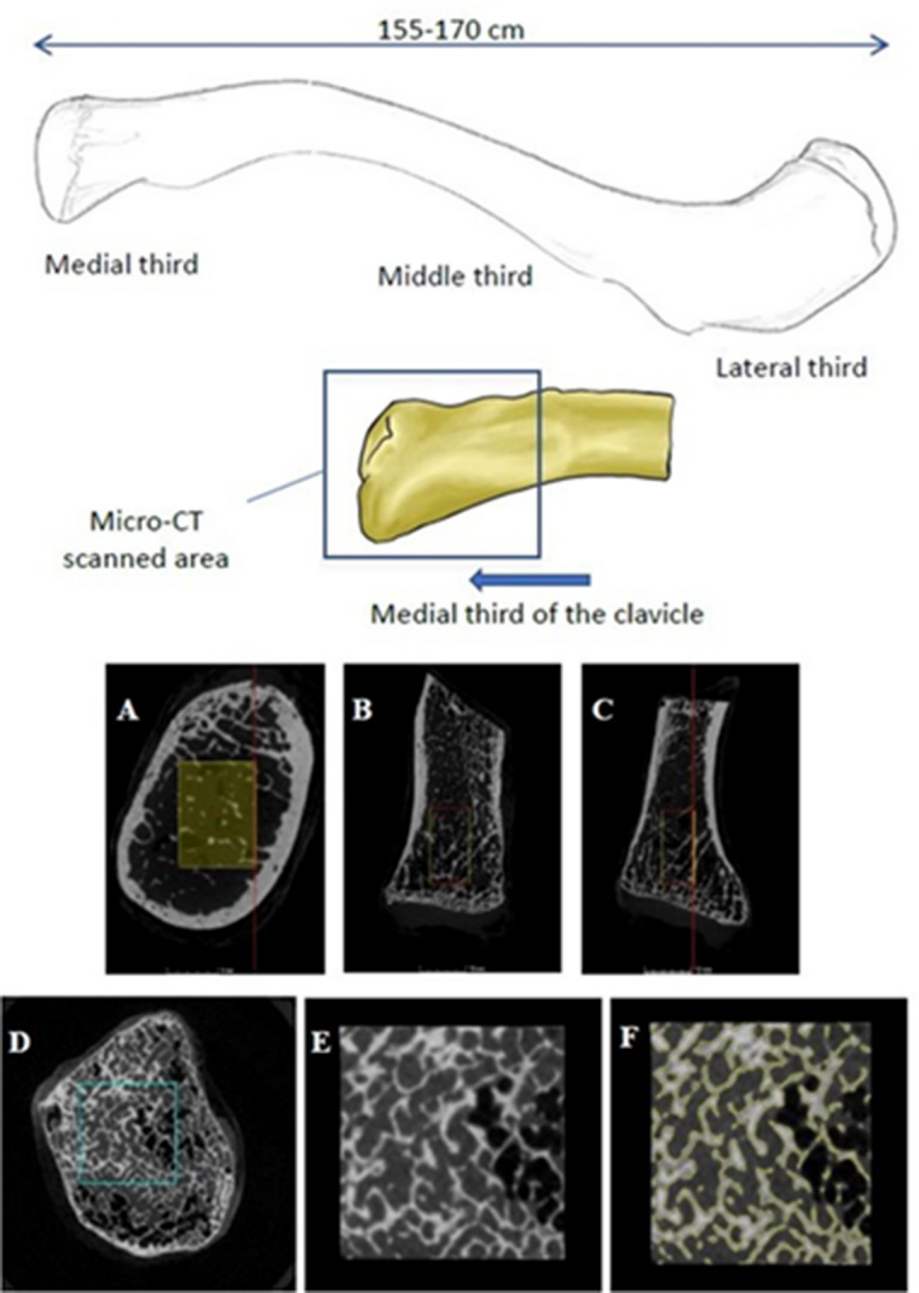
Shape and size of a human clavicle. **(A–C)** ROI selection process using VGStudio Max 2.1 representing maximum volume of trabeculae in the **(A)** transverse (x-y), **(B)** sagittal (y-z), and **(C)** coronal (x-z) planes. **(D–F)** Sequential process of surface determination for the VGStudio Max 2.1 software progressing through the **(D)** volume selection, **(E)** subsequent isolation of the ROI, and **(F)** segmentation of the bone material.

The samples (Figure 1) were scanned using a Nikon X-TEK CT H225 μ-CT cone beam scanner. Each specimen was imaged individually and aligned vertically within a cylindrical sample holder made of a non-attenuating material (ABS plastic) at 85 kV, 65 μA with a 500 ms exposure, and a voxel size of ~83 μm that met the recommendations of previous, peer-reviewed studies (Ding et al., 1999; Ding and Hvid, 2000; Bouxsein et al., 2010; Vale et al., 2013). The samples were imaged in air, rather than submersed in a liquid medium. The vertical alignment allowed images to be taken incrementally in a 360 degree rotation for three-dimensional reconstruction. The resulting image data was manually reconstructed using CTPro3D (XT 3.1.13) and examined for any visual distortion. Filters for beam hardening and noise reduction were applied during the reconstruction to produce images of the highest possible quality for the implemented testing protocol.

### Image Processing

Images were processed using VGStudio Max 2.1. A digital region of interest (ROI) was selected so as to reflect the maximum, or “bulk,” volume of trabecular bone in the medial epiphysis (Figures 1A–C). Some previous studies engaging in histomorphometry (Zioupos et al., 2008; Adams et al., 2018) have physically excised cores or cubed sections of trabecular bone from bones such as the proximal femur, vertebrae, or animal models where the quantity of cancellous bone is considerably higher and more likely to withstand the extraction process (Zioupos et al., 2008; Amson et al., 2017; Adams et al., 2018; Rieger et al., 2018; Vidal et al., 2018). Such excisions are invasive and possibly detrimental to the structural integrity of the trabecular network. The use of non-invasive scanning, on the other hand, is advantageous in forensics where the fragility of the sample and consent from the next of kin can greatly affect the extent of the analysis and choice of methods used (Stein and Granik, 1976; Stout and Paine, 1992). Furthermore, protocols which measure the “bulk” volume of trabecular bone have been considered more favorably of late (Salmon et al., 2015; Zhang et al., 2015; Amson et al., 2017; Vidal et al., 2018) compared to the use of a scaled, or constant volume. Following the isolation of the ROI for each specimen, a method for surface determination was applied to segment bone material from air by using a gray level threshold to digitally isolate the bone from the background (Figures 1D–F). This procedure is informed by previous studies from our laboratories which utilized the same software package (Adams et al., 2018; Greenwood et al., 2018).

**Figure 2:**
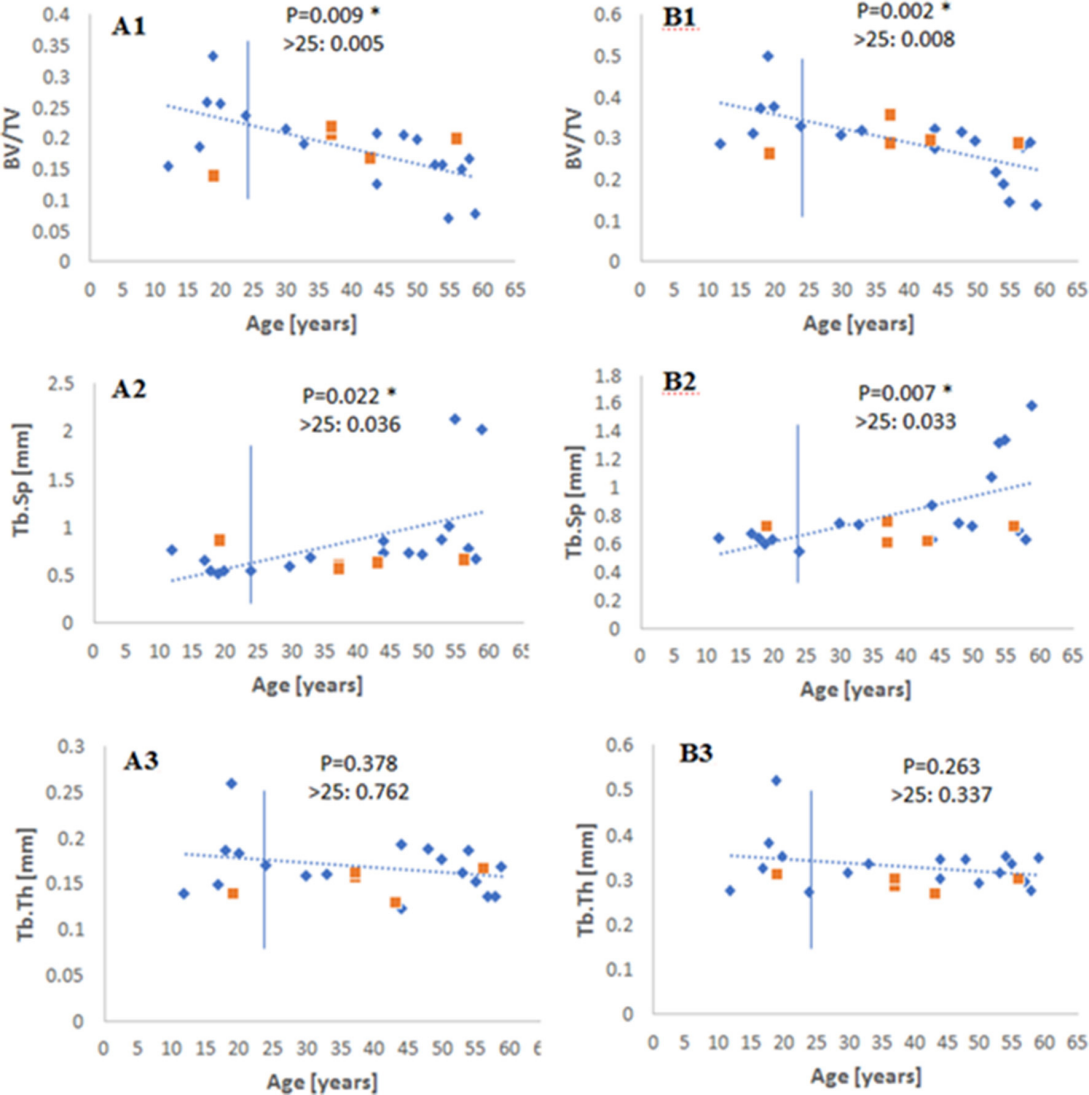
Scatterplot of BV/TV, Tb.Sp, and Tb.Th quantified using **(A1–A3)** VGStudio Max 2.1, and **(B1–B3)** BoneJ, across the age range. The vertical bar is at an age ~25 years when maximum skeletal height is reached. The level of statistical significance is shown for the whole range and for values older than 25 year old (Blue diamonds—males; orange squares—females).

The following morphometric indices were then generated: bone volume fraction (BV/TV), specific bone surface for a given volume (BS/BV), in addition to mean trabecular thickness (Tb.Th), number (Tb.N), and spacing (Tb.Sp). In addition, a measure of the connectivity, connectivity density (Conn.D) and degree of anisotropy (DA), which defines the preferred orientation of the trabecular bone in a given volume from highly isotropic (0) to anisotropic (1), were also performed using BoneJ (Doube et al., 2010), an open access ImageJ plug-in (Cui et al., 2008; Chen et al., 2013; Vale et al., 2013; Whitmarsh et al., 2019). In order to validate the results for BV/TV, Tb.Th and Tb.Sp, these measures were also calculated using BoneJ so the trends from both software packages could be compared. The comparison of the two analytical tools (VGStudio Max 2.1 vs. BoneJ) can be seen in Figure 2. The behavior was broadly similar, although different outcomes are always possible because of the different thresholding procedures set for each software package. The Structural Model Index (SMI), a morphometric parameter that is reported often, has not been included here due to acknowledged flaws and assumptions reported originally (Hildebrand and Rüegsegger, 1997), and since its inception (Viguet-Carrin et al., 2010; Salmon et al., 2015). This analytical process was repeated three times and a mean carried forward for each parameter generated for each individual.

### Mineral Density Calculation

A QRM μ-CT-HA calibration phantom was scanned using the same imaging protocol as the bone sample, and mean greyscale values were acquired from a ROI that did not exceed the confines of each of four (out of five) inserts with varying density (50, 200, 800, and 1,200 mg HA/cm^3^). The mean greyscale values were plotted against the corresponding calibrated density to generate a calibration curve. From this, along with the mean gray level value taken from the histogram for each specimen, the volumetric tissue mineral density (_v_TMD) was determined. Volumetric bone mineral density (_v_BMD) could then be calculated using the following equation (Meganck et al., 2009; Adams et al., 2018; Greenwood et al., 2018; Vidal et al., 2018; Whitmarsh et al., 2019)

(1) $$\begin{array}{r} \text{vBMD}=\text{vTMD x BV}/\text{TV} \end{array}$$

### Statistics

Boxplots were used to ascertain whether there were any outlying data points which may skew the data. Conditions were met to ensure a normal distribution of the data for the ensuing statistical tests. Regression analysis was then used to establish the significance of the relationships between each of the measured parameters and age. Henceforth, the threshold for statistical significance is *p* < 0.05, unless stated otherwise. The paucity of females in this sample means that these trend lines relate to structural changes in the cancellous bone of the males.

## Results

**Figure 3:**
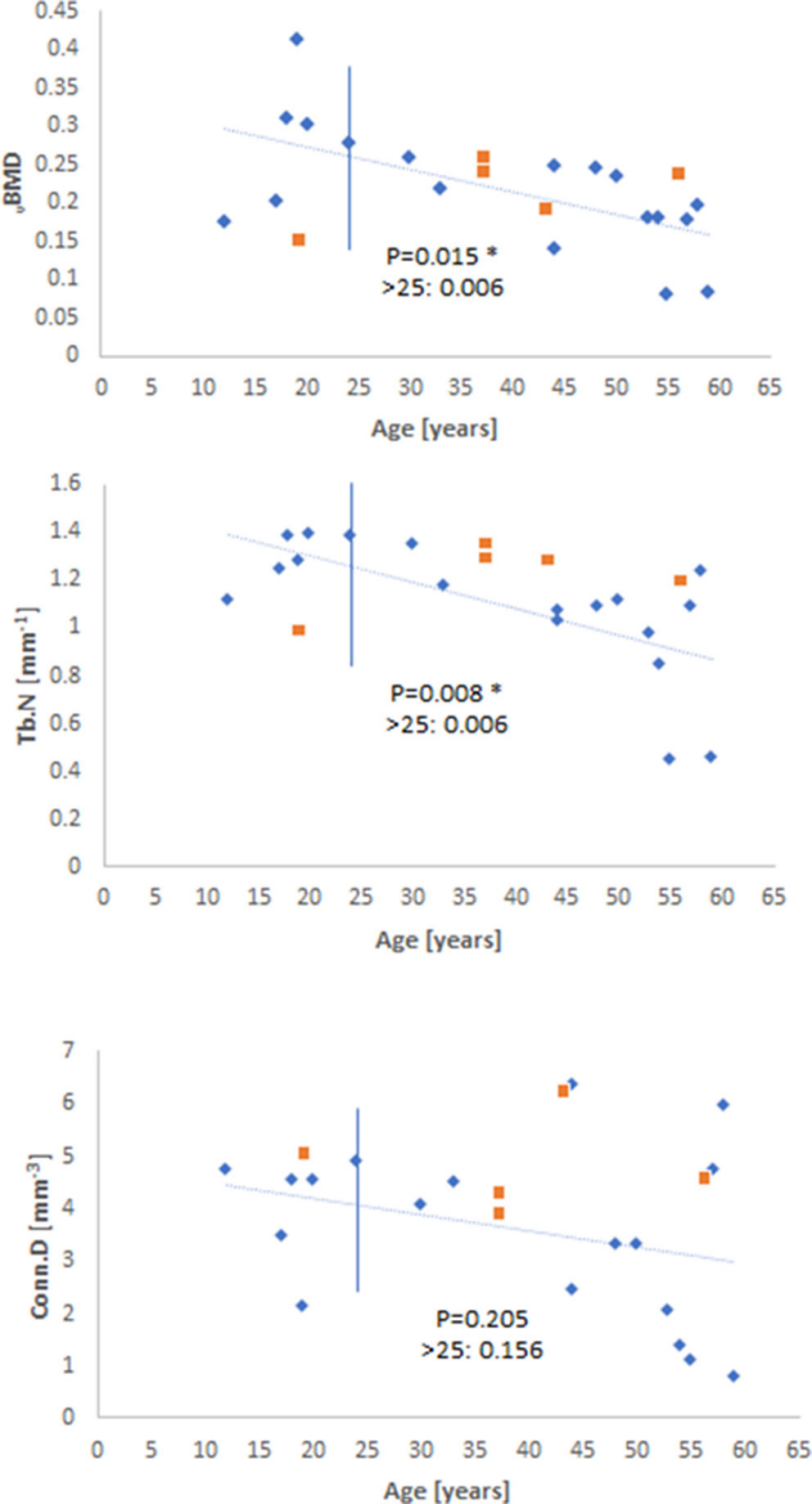
Scatterplots of vBMD, Tb.N, and Conn.D as a function of age. The level of statistical significance is shown for the whole range and for values older than 25 years old (Blue diamonds—males; orange squares—females).

**Figure 4:**
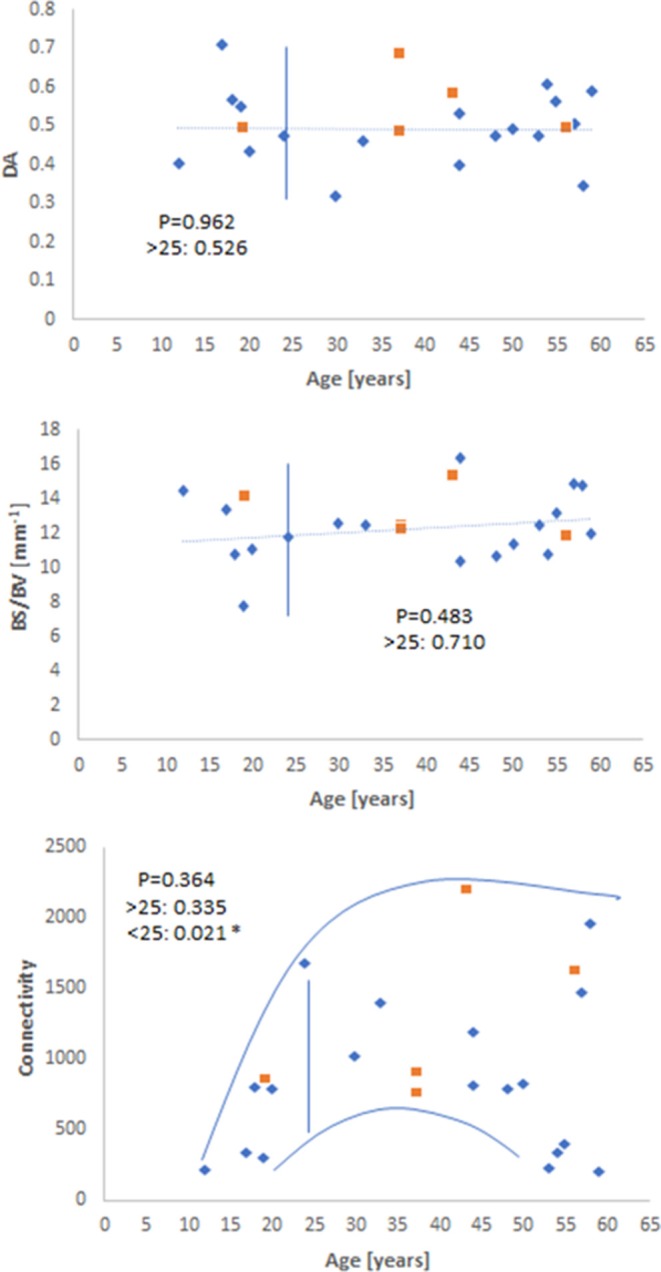
Scatterplot depicting the lack of change for DA and BS/BV as a function of age, in contrast to connectivity which exhibits two-phase behavior before- (significant increase with age) and after- skeletal maturity (slow decline with considerable value scatter) (Blue diamonds—males; orange squares—females).

A careful observation of the data showed that there was a qualitative change in behavior for before and after 23–25 years of age (when as we know maximum skeletal height is reached), so the data was examined for significance over the whole range and for samples older than 25 years. Measuring BV/TV, Tb.Sp, and Tb.Th by both VGStudio Max 2.1 and BoneJ showed similar trends in each method (Figure 2), both qualitatively and with regard to the level of statistical significance. When measured by BoneJ, BV/TV decreased with age by a rate of −0.009 ± 0.0033 per 5 years. The slightly different patterns and values in these two methodologies are most likely the result of different thresholding procedures set for each software package. Tb.Sp (Figure 2), as measured by both software packages, increased with age over the whole age range and for data after 25 years. Figure 2 also shows that Tb.Th remained constant throughout the ages (12–59 years) for this particular cohort. Figure 3 shows the behavior of _v_BMD and Tb.N which declined significantly with age over the whole range of ages and for after 25 years. Qualitatively, Conn.D decreases with age, but the non-significant result may be due to the scatter of the data (Figure 3). Figure 4 shows that BS/BV and DA displayed little association with age, while Connectivity showed a two-phase behavior, climbing before 25 years of age and slightly declining, but showing wide scatter, after 25 years.

## Discussion

Histomorphometric changes in the cancellous bone of regions in skeleton such as the decrease in BV/TV with age are well-established (Ding et al., 2002; Gong et al., 2005; Chen et al., 2008, 2010, 2013; Cui et al., 2008; Djuric et al., 2010; Thomsen et al., 2015; Whitmarsh et al., 2019). The loss of bone quantity is considered to be the most important factor affecting structural integrity with age, but “bone quality” including thinning, number, and connectivity of the trabeculae influence the mechanical competency of bone (Hildebrand et al., 1999; Compston, 2006; Djuric et al., 2010; Vale et al., 2013).

The decline in _v_BMD and Tb.N paralleled the decline in bone volume BV/TV in the current study which, coupled with the increase in Tb.Sp indicates perforation of, and presence of less densely connected trabeculae. These changes are in spite of the prolonged maturation period of the bone in question (clavicle), which may have delayed the onset of skeletal degeneration with age (Milenkovic et al., 2013). Furthermore, the decline in _v_BMD indicates changes to volumetric mineral density which may affect the ability of the material to resist deformation *in vivo* (Hunt and Donnelly, 2016). Alterations to some morphometric parameters but not others can provide further information about the underlying mechanisms which lead to structural changes with age. Tb.Th remained constant with age for this particular dataset, regardless of the software package used (VGStudio Max 2.1 *P* = 0.378; BoneJ *P* = 0.263), this is combined with a decrease in Tb.N (*P* = 0.008) suggesting that there may not be a compensatory thickening of the trabeculae to maintain load bearing capacity, but perhaps the removal of sections of the trabecular network which cannot withstand a particular load (Chen et al., 2008, 2010, 2013; Cui et al., 2008). The increase in Tb.Sp observed in this study reflects a consensus in the literature concerning the age-related change to the inter-trabecular space between the trabeculae (Chen et al., 2008, 2013), although this trend is reportedly more significant for other weight-bearing elements like the proximal femur and lumbar vertebrae which may have different remodeling rates to the clavicle (Cui et al., 2008; Chen et al., 2010, 2013). These observations support the notion that bone loses strength via the perforation and ultimate loss of bone volume rather than thinning of the trabecular struts (Parfitt et al., 1983; Parfitt, 1984; Chen et al., 2008, 2013; Cui et al., 2008).

A lack of change for DA in the clavicle (Figure 4) contrasts with the increase in DA reported in other studies (Ding et al., 2002; Gong et al., 2005; Cui et al., 2008; Djuric et al., 2010) for the proximal femur and tibia. However, in some cases (Chen et al., 2013; Whitmarsh et al., 2019), DA exhibits no significant trend with age, or this structural modification is not detected until later in life (Cui et al., 2008). For Cui et al. (2008), DA drastically changed after the seventh decade was surpassed, but remained largely unchanged until this stage. This is an age which exceeds the maximum for this study, and provides one possible explanation as to why no trend was detected for this sample, specifically, as well as the fact that the mechanical functionality of the clavicle is completely different from the femur and vertebrae (Martin, 1993).

An acknowledgeable limitation of the current study is the sample size. A larger and wider sample size would be needed to validate the findings presented here, and to monitor any changes to Tb.Th and DA that may occur in later life, which has been reported for other bone elements such as the proximal femur, proximal tibia, and lumbar vertebrae (Ding et al., 2002; Gong et al., 2005; Cui et al., 2008). Figures 2–4 also hint that there may be a qualitative age effect before- and after- 25 years. This is the age at which full skeletal height is reached. If that is the case, then complementary forensic identification methods can be used to first: (i) define whether a skeleton is pre- or post- 25 years, and then (ii) apply histomorphometry to tell the age of the unknown individual by more refined statistics on these bone parameters and perhaps using a generalized linear model like those used previously in our research group (Zioupos et al., 2014; Bonicelli et al., 2017).

## Conclusions

The findings presented in this study on the medial clavicle provide a basis for the future exploitation of this sub-region of the skeleton in forensic practice. This approach can be added to the usual macro-scopic biomarkers on the articular surface and the stages of ossification derived by common radiographic methods. The implementation of μ-CT: (1) allows the non-invasive quantification of trabecular bone housed inside the medial end of the clavicle, (2) where the tissue is protected in taphonomy and retains its architecture, (3) where it may survive intact in a crime scene; (4) where it may not will also not alter with time; (5) the μ-CT scan data can be produced relatively fast which is important in forensic cases; and (6) it can be used in micro-finite element models to perform simulations concerning the mechanical competency of bone in various loading scenarios. If supplemented by other techniques, such as characterization of micro-mechanical properties using nanoindentation or physicochemical analysis of the bone matrix it can potentially provide an age-at-death prediction model for both young (<25 years) and mature (>25 years) skeletons.

## Data Availability Statement

Datasets for this article are available through the Cranfield University CORD data depository and preservation system at https://cranfield.figshare.com (DOI: https://doi.org/10.17862/cranfield.rd.11673606.v1).

## Ethics Statement

This study involving human remains was reviewed and approved by ethical committees at the point of origin and at the point of analysis. Human cadaveric samples from 24 right clavicles dissected at autopsy (18 M/6 F) were provided by the forensic institute in Tirana, Albania. Ethical approval was provided by Cranfield University Ethics Committee (CURES/2294/2017), the Head of Control Department Investigation and Prosecution of the General Prosecutor, Ministry of Justice, Tirana, Albania and NHS tissue governance audit (ICA01/17). The authors are grateful to the various regulatory agencies for their input that allowed this study to take place. All samples were anonymised prior to acquisition.

## Author Contributions

HM, EK, and PZ: conceptualization and data curation. EK and PZ: formal analysis. PZ: funding acquisition, project administration, and resources. HM, BX, EK, and PZ: investigation. HM, CG, and PZ: methodology. HM and PZ: software. EK, NM-G, and PZ: supervision. EK: validation. HM: visualization and writing (original draft). HM, CG, BX, EK, NM-G, and PZ: writing (review and editing).

### Conflict of Interest

The authors declare that the research was conducted in the absence of any commercial or financial relationships that could be construed as a potential conflict of interest.

## References

[B1] Adams G. J. Cook R. B. Hutchinson J. R. Zioupos P. (2018). Bone apparent and material densities examined by cone beam computed tomography and the archimedes technique: comparison of the two methods and their results. Front. Mech. Eng. 3:23 10.3389/fmech.2017.00023

[B2] Amson E. Arnold P. Van Heteren A. H. Canoville A. Nyakatura J. A. (2017). Trabecular architecture in the forelimb epiphyses of extant xenarthrans (Mammalia). Front. Zool. 14:52. 10.1186/s12983-017-0241-x 29213295 PMC5707916

[B3] Beck B. Marcus R. (1999). Impact of physical activity on age-related bone loss, in The Aging Skeleton, eds Rosen C. J. Glowacki J. Bilezikian J. P (San Diego, CA: Academic Press), 467–478. 10.1016/B978-012098655-2/50041-7

[B4] Bonicelli A. Xhemali B. Kranioti E. F. Zioupos P. (2017). Rib biomechanical properties exhibit diagnostic potential for accurate ageing in forensic investigations. PLoS ONE 12:e0176785. 10.1371/journal.pone.0176785 28520764 PMC5435173

[B5] Bouxsein M. L. Boyd S. K. Christiansen B. A. Guldberg R. E. Jepsen K. J. Müller R. (2010). Guidelines for assessment of bone microstructure in rodents using micro-computed tomography. J. Bone Miner. Res. 25, 1468–1486. 10.1002/jbmr.141 20533309

[B6] Chen H. Shoumura S. Emura S. Bunai Y. (2008). Regional variations of vertebral trabecular bone microstructure with age and gender. Osteoporos. Int. 19, 1473–1483. 10.1007/s00198-008-0593-3 18330606

[B7] Chen H. Zhou X. Fujita H. Onozuka M. Kubo K. Y. (2013). Age-related changes in trabecular and cortical bone microstructure. Int. J. Endocrinol. 2013:213234. 10.1155/2013/213234 23573086 PMC3614119

[B8] Chen H. Zhou X. Shoumura S. Emura S. Bunai Y. (2010). Age- and gender-dependent changes in three-dimensional microstructure of cortical and trabecular bone at the human femoral neck. Osteoporos. Int. 21, 627–636. 10.1007/s00198-009-0993-z 19543764

[B9] Compston J. (2006). Bone quality: what is it and how is it measured? Arq. Bras. Endocrinol. Metabol. 50, 579–585. 10.1590/S0004-27302006000400003 17117283

[B10] Compston J. (2011). Age-related changes in bone remodelling and structure in men: histomorphometric studies. J. Osteoporos. 2011:108324. 10.4061/2011/108324 22132344 PMC3206334

[B11] Crowder C. Pfeiffer S. (2010). The application of cortical bone histomorphometry to estimate age at death, in Age Estimation of the Human Skeleton, eds Latham K. E. Finnegan M (Springfield, IL: Charles C Thomas), 193–215.

[B12] Cui W. Q. Won Y. Y. Baek M. H. Lee D. H. Chung Y. S. Hur J. H.. (2008). Age-and region-dependent changes in three-dimensional microstructural properties of proximal femoral trabeculae. Osteoporos. Int. 19, 1579–1587. 10.1007/s00198-008-0601-7 18437273

[B13] Currey J. D. (2002). Bones: Structure and Mechanics. Princeton, NJ: Princeton University Press.

[B14] Ding M. Hvid I. (2000). Quantification of age-related changes in the structure model type and trabecular thickness of human tibial cancellous bone. Bone 26, 291–295. 10.1016/S8756-3282(99)00281-1 10710004

[B15] Ding M. Odgaard A. Hvid I. (1999). Accuracy of cancellous bone volume fraction measured by micro-CT scanning. J. Biomech. 32, 323–326. 10.1016/S0021-9290(98)00176-6 10093033

[B16] Ding M. Odgaard A. Linde F. Hvid I. (2002). Age-related variations in the microstructure of human tibial cancellous bone. J. Orthop. Res. 20, 615–621. 10.1016/S0736-0266(01)00132-2 12038639

[B17] Djuric M. Djonic D. Milovanovic P. Nikolic S. Marshall R. Marinkovic J.. (2010). Region-specific sex-dependent pattern of age-related changes of proximal femoral cancellous bone and its implications on differential bone fragility. Calcif. Tissue Int. 86, 192–201. 10.1007/s00223-009-9325-8 20012269

[B18] Doube M. Kłosowski M. M. Arganda-Carreras I. Cordelières F. P. Dougherty R. P. Jackson J. S.. (2010). BoneJ: free and extensible bone image analysis in ImageJ. Bone 47, 1076–1079. 10.1016/j.bone.2010.08.023 20817052 PMC3193171

[B19] Doyle E. Márquez-Grant N. Field L. Holmes T. Arthurs O. J. van Rijn R. R. (2019). Guidelines for best practice: imaging for age estimation in the living. J. Forensic Radiol. Imaging 16, 38–49. 10.1016/j.jofri.2019.02.001

[B20] Eckstein F. Matsuura M. Kuhn V. Priemel M. Müller R. Link T. M.. (2007). Sex differences of human trabecular bone microstructure in aging are site-dependent. J. Bone Miner. Res. 22, 817–824. 10.1359/jbmr.070301 17352643

[B21] Franklin D. (2010). Forensic age estimation in human skeletal remains: current concepts and future directions. Legal Med. 12, 1–7. 10.1016/j.legalmed.2009.09.001 19853490

[B22] Gong H. Zhang M. Yeung H. Y. Qin L. (2005). Regional variations in microstructural properties of vertebral trabeculae with aging. J. Bone Miner. Metab. 23, 174–180. 10.1007/s00774-004-0557-4 15750697

[B23] Greenwood C. Clement J. Dicken A. Evans P. Lyburn I. Martin R. M.. (2018). Age-related changes in femoral head trabecular microarchitecture. Aging Dis. 9, 976–987. 10.14336/AD.2018.0124 30574411 PMC6284768

[B24] Hildebrand T. Laib A. Müller R. Dequeker J. Rüegsegger P. (1999). Direct three-dimensional morphometric analysis of human cancellous bone: microstructural data from spine, femur, iliac crest, and calcaneus. J. Bone Miner. Res. 14, 1167–1174. 10.1359/jbmr.1999.14.7.1167 10404017

[B25] Hildebrand T. Rüegsegger P. (1997). Quantification of bone microarchitecture with the structure model index. Comput. Methods Biomech. Biomed. Engin. 1, 15–23. 10.1080/01495739708936692 11264794

[B26] Hunt H. B. Donnelly E. (2016). Bone quality assessment techniques: geometric, compositional, and mechanical characterization from macroscale to nanoscale. Clin. Rev. Bone Miner. Metabo. 14, 133–149. 10.1007/s12018-016-9222-4 28936129 PMC5604329

[B27] Johannesdottir F. Bouxsein M.L. (2018). Overview of bone structure and strength, in Genetics of Bone Biology and Skeletal Disease, 2nd Edn. eds Thakker R. V. Whyte M. P. Eisman J. A. Igarashi T (London: Academic Press), 197–208. 10.1016/B978-0-12804182-6.00012-5

[B28] Kellinghaus M. Schulz R. Vieth V. Schmidt S. Schmeling A. (2010). Forensic age estimation in living subjects based on the ossification status of the medial clavicular epiphysis as revealed by thin-slice multidetector computed tomography. Int. J. Legal Med. 124, 149–154. 10.1007/s00414-009-0398-8 20013127

[B29] LeBel M.-E. (2015). Clavicle fractures, in Shoulder and Elbow Trauma and its Complications, ed Greiwe M (Cambridge, UK: Elsevier Ltd), 191–213. 10.1016/B978-1-78242-449-9.00009-1

[B30] Lochmüller E. M. Matsuura M. Bauer J. Hitzl W. Link T. M. Müller R.. (2008). Site-specific deterioration of trabecular bone architecture in men and women with advancing age. J. Bone Miner Res. 23, 1964–1973. 10.1359/jbmr.080709 18665791

[B31] Martin B. (1993). Aging and strength of bone as a structural material. Calcif. Tissue Int. 53, S34–S40. 10.1007/BF01673400 8275378

[B32] Meganck J. A. Kozloff K. M. Thornton M. M. Broski S. M. Goldstein S. A. (2009). Beam hardening artifacts in micro-computed tomography scanning can be reduced by X-ray beam filtration and the resulting images can be used to accurately measure BMD. Bone 45, 1104–1116. 10.1016/j.bone.2009.07.078 19651256 PMC2783193

[B33] Milenkovic P. Djukic K. Djonic D. Milovanovic P. Djuric M. (2013). Skeletal age estimation based on medial clavicle—a test of the method reliability. Int. J. Legal Med. 127, 667–676. 10.1007/s00414-012-0791-6 23329360

[B34] Müller R. Rüegsegger P. (1997). Micro-tomographic imaging for the nondestructive evaluation of trabecular bone architecture, in Bone Research in Biomechanics, eds Lowet G. Ruegsegger P. Weinans H. Meunier A (Amsterdam: IOS Press, 61–79. 10168883

[B35] Müller R. Van Campenhout H. Van Damme B. Van Der Perre G. Dequeker J. Hildebrand T.. (1998). Morphometric analysis of human bone biopsies: a quantitative structural comparison of histological sections and micro-computed tomography. Bone 23, 59–66. 10.1016/S8756-3282(98)00068-4 9662131

[B36] Parfitt A. M. (1984). Age-related structural changes in trabecular and cortical bone: cellular mechanisms and biomechanical consequences. Calcif. Tissue Int. 36, S123–S128. 10.1007/BF02406145 6430512

[B37] Parfitt A. M. Mathews C. H. E. Villanueva A. R. Kleerekoper M. Frame B. Rao D. S. (1983). Relationships between surface, volume, and thickness of iliac trabecular bone in aging and in osteoporosis: implications for the microanatomic and cellular mechanisms of bone loss. Am. Soc. Clin. Invest. 72, 1396–1409. 10.1172/JCI111096 6630513 PMC370424

[B38] Rho J. Y. Kuhn-Spearing L. Zioupos P. (1998). Mechanical properties and the hierarchical structure of bone. Med. Eng. Phys. 20, 92–102. 10.1016/S1350-4533(98)00007-1 9679227

[B39] Rieger R. Auregan J. C. Hoc T. (2018). Micro-finite-element method to assess elastic properties of trabecular bone at micro- and macroscopic level. Morphologie 102, 12–20. 10.1016/j.morpho.2017.07.175 28893491

[B40] Rudolf E. Kramer J. Schmidt S. Vieth V. Winkler I. Schmeling A. (2018). Intraindividual incongruences of medially ossifying clavicles in borderline adults as seen from thin-slice CT studies of 2595 male persons. Int. J. Legal Med. 132, 629–636. 10.1007/s00414-017-1694-3 28944440

[B41] Salmon P. L. Ohlsson C. Shefelbine S. J. Doube M. (2015). Structure model index does not measure rods and plates in trabecular bone. Front. Endocrinol. 6:162 10.3389/fendo.2015.00162 PMC4602154 26528241

[B42] Schmeling A. Schulz R. Reisinger W. Mühler M. Wernecke K. D. Geserick G. (2004). Studies on the time frame for ossification of the medial clavicular epiphyseal cartilage in conventional radiography. Int. J. Legal Med. 118, 5–8. 10.1007/s00414-003-0404-5 14534796

[B43] Stein I. D. Granik G. (1976). Rib structure and bending strength: an autopsy study. Calcif. Tissue Res. 20, 61–73. 10.1007/BF02546398 1260494

[B44] Stout S. D. Paine R. R. (1992). Histological age estimation using rib and clavicle. Am. J. Phys. Anthropol. 87, 111–115. 10.1002/ajpa.1330870110 1736669

[B45] Thomsen J. S. Jensen M. V. Niklassen A. S. Ebbesen E. N. Brüel A. (2015). Age-related changes in vertebral and iliac crest 3D bone microstructure—differences and similarities. Osteoporos. Int. 26, 219–228. 10.1007/s00198-014-2851-x 25164697

[B46] Thomsen J. S. Niklassen A. S. Ebbesen E. N. Brüel A. (2013). Age-related changes of vertical and horizontal lumbar vertebral trabecular 3D bone microstructure is different in women and men. Bone 57, 47–55. 10.1016/j.bone.2013.07.025 23899636

[B47] Tranquilli Leali P. Doria C. Zachos A. Ruggiu A. Milia F. Barca F. (2009). Bone fragility: current reviews and clinical features. Clin. Cases Miner Bone Metab. 6, 109–113. 22461157 PMC2781230

[B48] Ulrich D. van Rietbergen B. Laib A. Rüegsegger P. (1999). The ability of three-dimensional structural indices to reflect mechanical aspects of trabecular bone. Bone 25, 55–60. 10.1016/S8756-3282(99)00098-8 10423022

[B49] Vale A. C. Pereira M. F. C. Maurício A. Vidal B. Rodrigues A. Caetano-Lopes J. (2013). Micro-computed tomography assessment of human femoral trabecular bone for two disease groups (fragility fracture and coxarthrosis): age and gender related effects on the microstructure. J. Biomed. Sci. Eng. 6, 175–184. 10.4236/jbise.2013.62021

[B50] Vidal B. Cascao R. Finnila M. A. J. Lopes I. P. Saarakkala S. Zioupos P.. (2018). Early arthritis induces disturbances at bone nanostructural level reflected in decreased tissue hardness in an animal model of arthritis. PLoS ONE 13:e0190920. 10.1371/journal.pone.0190920 29315314 PMC5760022

[B51] Viguet-Carrin S. Follet H. Gineyts E. Roux J. P. Munoz F. Chapurlat R.. (2010). Association between collagen cross-links and trabecular microarchitecture properties of human vertebral bone. Bone 46, 342–347. 10.1016/j.bone.2009.10.001 19836004

[B52] Whitmarsh T. Otake Y. Uemura K. Takao M. Sugano N. Sato Y. (2019). A cross-sectional study on the age-related cortical and trabecular bone changes at the femoral head in elderly female hip fracture patients. Sci. Rep. 9:305. 10.1038/s41598-018-36299-y 30670734 PMC6343024

[B53] Wittschieber D. Ottow C. Schulz R. Püschel K. Bajanowski T. Ramsthaler F.. (2016). Forensic age diagnostics using projection radiography of the clavicle: a prospective multi-center validation study. Int. J. Legal Med. 130, 213–219. 10.1007/s00414-015-1285-0 26518299

[B54] Wittschieber D. Schulz R. Vieth V. Küppers M. Bajanowski T. Ramsthaler F.. (2014). The value of sub-stages and thin slices for the assessment of the medial clavicular epiphysis: a prospective multi-center CT study. Forensic Sci. Med. Pathol. 10, 163–169. 10.1007/s12024-013-9511-x 24277267

[B55] Zhang R. Gong H. Zhu D. Ma R. Fang J. Fan Y. (2015). Multi-level femoral morphology and mechanical properties of rats of different ages. Bone 76, 76–87. 10.1016/j.bone.2015.03.022 25857690

[B56] Zhou Q. Rouhana S. W. Melvin J. W. (1996). Age effects on thoracic injury tolerance, in Stapp Car Crash Conference Proceedings (Albuquerque, NM), 137–148. 10.4271/962421

[B57] Zioupos P. Cook R. B. Hutchinson J. R. (2008). Some basic relationships between density values in cancellous and cortical bone. J. Biomech. 41, 1961–1968. 10.1016/j.jbiomech.2008.03.025 18501911

[B58] Zioupos P. Williams A. Christodoulou G. Giles R. (2014). Determining “age at death” for forensic purposes using human bone by a laboratory-based biomechanical analytical method. J. Mech. Behav. Biomed. Mater. 33, 109–123. 10.1016/j.jmbbm.2013.10.015 24286969

